# Diagnostic performance of deep-learning-based screening methods for diabetic retinopathy in primary care—A meta-analysis

**DOI:** 10.1371/journal.pone.0255034

**Published:** 2021-08-10

**Authors:** Larisa Wewetzer, Linda A. Held, Jost Steinhäuser

**Affiliations:** University Medical Center Schleswig-Holstein, Institute for Family Medicine, Lubeck, Germany; Universidad Miguel Hernandez de Elche, SPAIN

## Abstract

**Background:**

Diabetic retinopathy (DR) affects 10–24% of patients with diabetes mellitus type 1 or 2 in the primary care (PC) sector. As early detection is crucial for treatment, deep learning screening methods in PC setting could potentially aid in an accurate and timely diagnosis.

**Purpose:**

The purpose of this meta-analysis was to determine the current state of knowledge regarding deep learning (DL) screening methods for DR in PC.

**Data sources:**

A systematic literature search was conducted using Medline, Web of Science, and Scopus to identify suitable studies.

**Study selection:**

Suitable studies were selected by two researchers independently. Studies assessing DL methods and the suitability of these screening systems (diagnostic parameters such as sensitivity and specificity, information on datasets and setting) in PC were selected. Excluded were studies focusing on lesions, applying conventional diagnostic imaging tools, conducted in secondary or tertiary care, and all publication types other than original research studies on human subjects.

**Data extraction:**

The following data was extracted from included studies: authors, title, year of publication, objectives, participants, setting, type of intervention/method, reference standard, grading scale, outcome measures, dataset, risk of bias, and performance measures.

**Data synthesis and conclusion:**

The summed sensitivity of all included studies was 87% and specificity was 90%. Given a prevalence of DR of 10% in patients with DM Type 2 in PC, the negative predictive value is 98% while the positive predictive value is 49%.

**Limitations:**

Selected studies showed a high variation in sample size and quality and quantity of available data.

## Introduction

The prevalence of diabetes mellitus (DM) type 1 and 2 is rising, with approximately 629 million people worldwide expected to be suffering from this disease by the year 2045. In Germany, the number of patients with DM currently amounts to 7.5 million [1]. Diabetic retinopathy (DR) constitutes one of the most common serious complications of DM and affects approximately 25–35% of these patients [2]. It is defined as damage to the blood vessels of the eyes caused by high blood glucose levels. The prevalence of this complication in Germany ranges from 10% to 30%, depending on the health care sector. In primary care (PC), 24% of type 1 and 10% of type 2 DM patients are reportedly diagnosed with a DR, while in secondary and tertiary care the reported prevalence is higher (type 1 DM: 27–30%, type 2 DM: 20–25%) [3].

If untreated, DR may cause visual impairments or even blindness [4]. According to a well-used classification, the Early Treatment of Diabetic Retinopathy Study (EDTRS) severity scale, different stages of diabetic retinopathy can be differentiated: non-proliferative stages, a proliferative stage and diabetic macular edema. The last-named stage represents an accumulation of fluid in the retina and is the major cause of decreased vision for patients suffering from diabetic retinopathy [5]. However, the early stages of the disease remain asymptomatic for most patients. Therefore, within the framework of a screening, it is essential to detect any early sign of the disease to prevent more serious complications.

Currently available therapies, include laser-assisted photocoagulation and application of intravitreal medication and can potentially prevent loss of vision if applied in the early stages of the disease [6]. The aim of these methods is to stabilize the remaining intact retina cells to retain vision, yet already damaged retinal cells cannot be restored with these tools [7].

Experiences as during the current COVID-19 pandemic show that treatments, which are not considered an emergency might get shifted, access to health care providers can sometimes be limited and consequently check-ups delayed.

According to the national guideline ("Nationale Versorgungsleitlinie Typ-2-Diabetes: Therapie"), the patient with DM is responsible to maintain regular ophthalmological check-ups, while in the absence of a diagnosed retinopathy, the PC provider or diabetologist is responsible [7]. In contrast to the importance of regular screenings, approximately 20–40% of these patients do not meet the recommended screening intervals [8], with only an estimated 45–50% of eye exams indicated for patients with DM actually taking place [9]. Part of this problem is a lack of adherence to regular eye screenings which may potentially be improved by using information technology tools. Such tools might improve the accessibility of health care and enable physicians to offer individualized screenings that are tailored to the risk of developing a retinopathy. This in turn could improve the willingness of patients with DM to participate in such screenings, as DM management is primarily accomplished by PC providers [8]. In addition, 90% of German citizens are in contact with their PC provider at least once a year [10]. Artificial intelligence as a tool of information technology may be suitable in optimizing the interaction between patients and PC providers. Artificial intelligence is defined as an intelligent device that can perceive their environment and react to it to achieve a defined goal [11]. Deep learning (DL) is a form of artificial intelligence that represents an algorithm for artificial neuronal networks with multiple layers [12]. DL refers to a class of machine learning algorithms that is based on the combination of multiple layers of different types of artificial neural networks Such convolutional neural networks can differentiate between different objects or aspects of an image. Some DL tools are employed in healthcare already, e.g. in the screening of histopathological slides for cancer detection or evaluation of radiographs [13, 14]. Whether DL systems could be meaningfully employed in the screening of patients with DM for retinopathy to optimize the current screening strategy, is yet unknown. Nonetheless, it is feasible to assume that DL algorithms could be employed to evaluate images of the fundus without mydriasis, a process that might be learned and performed by any medical personnel. In order to allow for the clinical application of such a tool, the algorithm must be able to distinguish between healthy patients and those with retinopathy. This may be assessed based on the sensitivity and the specificity of the test according to a defined threshold and receiver operating characteristics curves.

The aim of this meta-analysis was to determine the current state of knowledge regarding the implementation of DL screening methods in PC and to analyze the suitability of such systems in DR diagnosis.

## Methods

This systematic meta-analysis was conducted according to the criteria specified in the „Preferred Reporting Items for Systematic Review and Metaanalysis of Diagnostic Test Accuracy”[15].

### Data sources and searches

The PICO scheme (Patient/Population/Problem, Intervention, Control/Comparison, Outcome) was used to define the research question and determine appropriate search terms [16]. The search strategy was designed to combine several suitable search terms with the aim to gain a comprehensive insight into the available literature. The search algorithm was as follows: (("Primary Health Care” OR "General Practice" OR "Comprehensive Health Care" OR "Family Practice" OR "Ambulatory Care" OR "Physicians, Primary Care" OR "Primary Care Nursing") AND ("Artificial Intelligence" OR "Machine Learning" OR "Deep Learning" OR "Diagnosis, Computer-Assisted" OR "Smartphone" OR "Decision Support Systems, Clinical" OR "Image Processing, Computer-Assisted" OR "Neural Networks, Computer") AND ("Diabetic Retinopathy")).

The algorithm was employed on March 12, 2020 in a systematic literature search using the electronic databases Medline, Web of Science, and Scopus to identify suitable studies. Due to the comparatively rapid development of this research field the search was limited to articles published in 2015 or later. We also searched the reference lists of retrieved studies and the opengrey database (http://www.opengrey.eu/) to identify additional studies missed in the systematic search. No eligible studies were found.

### Inclusion criteria

Included were studies assessing deep learning methods in the context of DR detection and the suitability of these screening systems (diagnostic parameters including area under the curve/AUC, sensitivity and specificity, information on datasets, setting and number of images) in a PC setting. There was no differentiation between studies assessing patients with type 1 or type 2 diabetes. Only studies published in English were included.

### Exclusion criteria

Excluded were studies focusing on lesions, applying conventional diagnostic imaging tools, or those conducted in secondary or tertiary care. All publication types other than original research studies on human subjects (i.e., reviews, editorials, conference abstracts, animal studies) were excluded.

### Study selection

Suitable studies were selected based on the inclusion and exclusion criteria using the Covidence review management software in a 2-step-procedure by two researchers (LH und LW) independently. In the first step, titles and abstracts of the search results were screened, followed by full text evaluation of studies deemed potentially suitable in the first step. Any discrepancies between the two researchers were solved through discussion with a third researcher (JS).

### Data extraction and quality assessment

The following data was extracted from included studies: authors, title, year of publication, objectives, participants, setting, type of intervention/method, reference standard, grading scale, outcome measures, dataset, risk of bias, and performance measures (area under the curve (AUC), sensitivity, 1-specificity). The study quality was evaluated using Quality Assessment of Diagnostic Accuracy Studies (QUADAS), a tool to assess the quality of studies to be included in a systematic review [17]. Data extraction and quality assessment were performed independently by two researchers according to a pre-defined assessment scheme.

The quality of the selected studies was evaluated based on their internal and external validity. Criteria to assess the internal validity included the consideration of a valid reference standard, blinded evaluation of the index and reference tests, avoidance of bias with regards to verification, study design, and non-clinical analysis of the index tests. Different sources of bias had to be excluded in order to achieve high internal validity. These pertain to spectrum bias (influence of patient spectrum and disease severity), differential verification bias (use of different reference tests depending on results of the index test), diagnostic review bias (influence of index test results on final diagnosis), and incorporation bias (influence of index test results on reference test). Criteria to evaluate the external validity include disease spectrum, setting, previous tests, referral filters, duration of disease before diagnosis, comorbidities, demographics, conduction of the index tests, relative proportion of missing data, and reproducibility of the index test.

### Data synthesis and analysis

Performance data (sensitivity and specificity) was subjected to meta-analysis, and forest plots were generated to compare the performance of the systems in the detection of DR. In this analysis, different types of DR reported in the studies (any DR, rDR, vtDR) were pooled as any DR because as part of the screening the differentiation between DR present, yes or no, is of relevance, while the differentiation between different types is at this stage not relevant. In addition, the focus in the PC setting is on timely detection of DR ideally in the early stages, rather than later or chronic stages. Meta-Disc 1.4 software was used to conduct a statistical analysis and to visualize the pooled results on sensitivity, specificity, SROC and diagnostic odds ratio (DOR). Initially, quantitative data from the selected studies was summarized and tested for statistical heterogeneity. Reasons for methodological and clinical heterogeneity were evaluated. Pooled effect estimates and DOR were calculated to judge on the suitability of the test to allow for assessment of the results. I^2^ was calculated to determine the relation between the variance of individual studies and the overall variance calculated in the meta-analysis. To analyze the variance of individual effect estimates, Cochrane’s Q was determined. Chi^2^ was calculated to assess the variance between the individual studies. A funnel plot was generated to assess publication bias.

## Results

### Included studies

**Fig 1:**
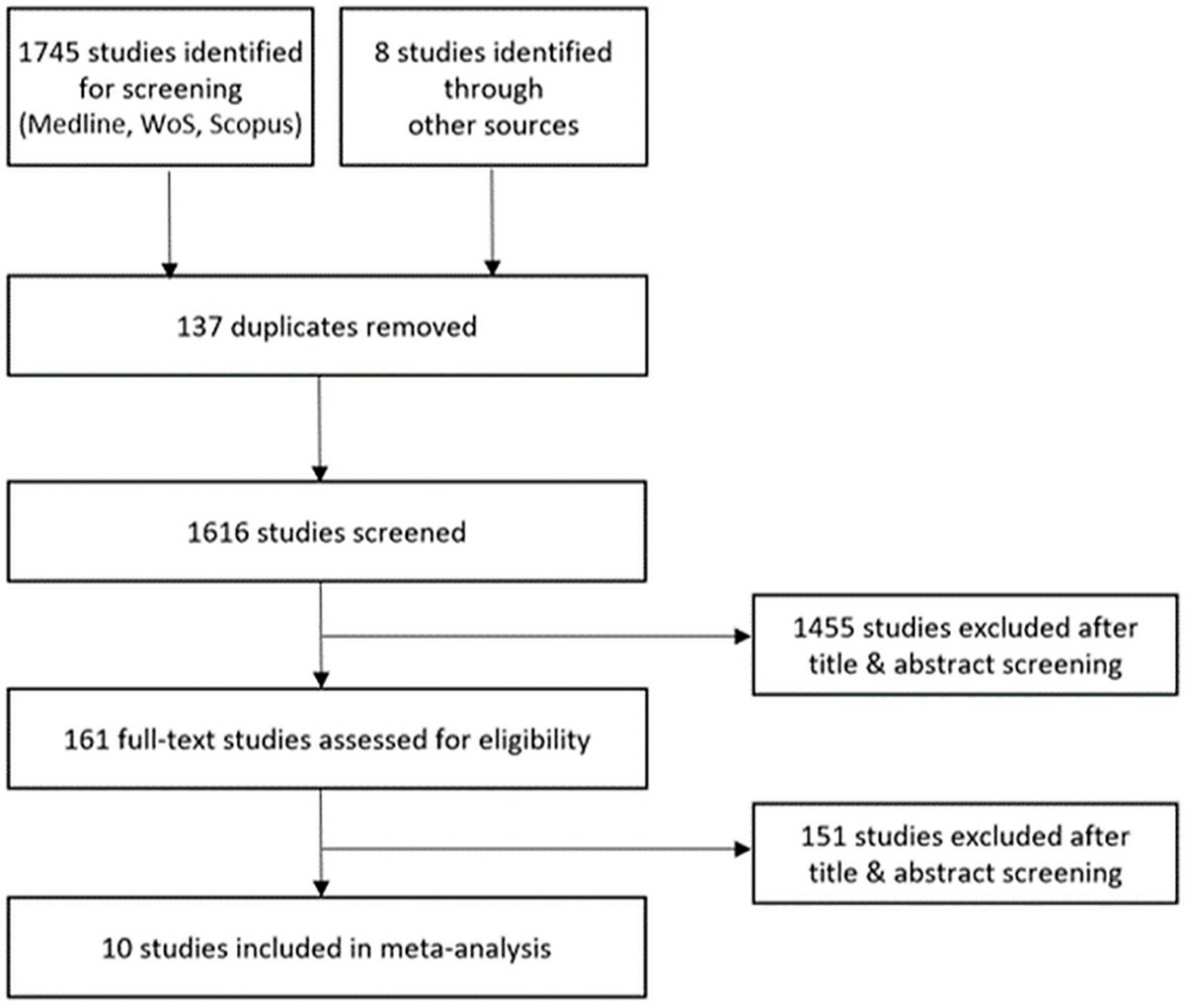
Flowchart of the 2-step study selection.

The results of the 2-step study selection process are shown in the flowchart in Fig 1.

In total, 10 studies were identified and included in the meta-analysis. Among these are studies with clinically tested devices with registration for PC as well as devices registered for PC that were tested on publicly available datasets, non-certified, clinically tested DL algorithms, and DL algorithms tested on publicly available datasets.

Abràmoff et al. (2018) conducted an observational analysis of 900 patients with DM at ten PC sites, with 819 participants suitable for analysis [18]. The intervention consisted of implementation of an IDx-DR autonomous artificial intelligence system, in which the detectors were implemented as multilayer CNN and DR graded according to the Early Treatment Diabetic Retinopathy Severity Scale. The outcome measure was the automated detection of DR and macular edema. 23.8% of the images showed a DR/macular edema, with a sensitivity of 87.2% and a specificity of 90.7%. Natarajan et al. (2019) analyzed the performance of an automated smartphone-based CNN system to detect rDR and determined a rDR prevalence of 12.21%, with a sensitivity of 100.0% and a specificity of 88.4%. For any DR the sensitivity and specificity was 85,2% and 92% [19]. In the retrospective study by Verbraak et al. (2019) over 1.600 patients with type 2 DM were screened for DR at a PC center using the IDx-DR-EU-2.1 system with a Topcon TRC-NW200 camera [20]. They discovered a prevalence of 11.7% of mild DR, 3.9% of moderate DR and 1.1 of vtDR, with a sensitivity of 100% for vtDR and 79.4% for more than mild DR. The specificity reached 97.8% for vtDR and 93.8% for more than mild DR. Walton et al. (2016) assessed over 15.000 patients with DM (types 1 and 2) for DR using the Intelligent Retinal Imaging System (IRIS) and a CenterVue camera to obtain the images [21]. The outcome was the identification of sight-threatening eye disease that was present in 4.72% of patients. The sensitivity to detect vtDR using this system was 66.4% and the specificity 72.8%.

Bhaskaranand et al. (2019) retrospectively evaluated the suitability of the EyeArt system, a cloud-based artificial intelligence device, on a large dataset of over 100.000 patients with diabetes [22]. rDR was detected in 19.3% of these patients, with a sensitivity of 91.3%, a specificity of 91.1%, and an AUC of 0.965.

Bellemo et al. (2019) analyzed the value of a mobile diabetic retinopathy screening in Zambia using a digital retinopathy system fundus camera taking 45° retinal fundus images. The artificial intelligence model consisted of two combined CNN and the outcome was differentiated by rDR, vtDR and DME. In total, 4.504 images were evaluated. The prevalence of rDR was 22.5%, that of vtDR 5.5%, while DME was detected in 8.1% of patients. rDR detection showed a sensitivity of 92.25% and a specificity of 89.04%, with an AUC of 0.973. The sensitivity of vtDR detection was determined as 99.42% (AUC: 0.934), that of DME as 97.19% (AUC: 0.942). Kanagasingam et al. (2018) recruited 193 patients with diabetes to evaluate an artificial intelligence-based CNN using a Topcon fundus camera to obtain the images [23]. 10 of the 193 patients (5.18%) had DR, with a sensitivity of 100% and a specificity of 92%. Ting et al. (2017) employed a CNN-based deep learning system on 76.370 images of patients with diabetes in Singapore and found a prevalence of 8.0% for any DR, 3.0% for rDR and 0.6% for vtDR. The calculated sensitivity was 90.5% and 100% for rDR and vtDR, respectively, and the specificity was 91.6% and 91.1% [24]. The AUC for rDR was determined at 0.936, for vtDR at 0.958.

Gulshan et al. (2016) retrospectively validated the EyePACS (9.963 images) and Messidor (1.748 images) systems using the DL algorithm [25]. rDR was detected in 7.8% of the images using EyePACS and in 14.6% of images using Messidor. The sensitivity and specificity were assessed at two operating cut points, one with a high specificity, and the other with a high sensitivity. For the first cut point, the sensitivity was 90.3% (EyePACS) and 87% (Messidor), for the second cut-point it was calculated as 97.5% (EyePACS) and 96.1% (Messidor). The calculated specificity amounted to 98.1% (EyePACS) and 98.5% (Messidor) at the first cut point, and to 93.4% (EyePACS) and 93.9% (Messidor) at the second cut point. For EyePACS, the calculated AUC was 0.991 for rDR, while it was 0.990 for Messidor. Raju et al. (2017) employed different fundus cameras to test the deep learning application EyePACS to categorize the stage of diabetic retinopathy in a community-based setting [26]. More than 50,000 images were evaluated with the following results: no DR was detected in 73.79% of the samples, mild npDR in 7.02% of the samples, moderate npDR in 14.67% of the samples, and severe npDR in 2.48% of the samples. Proliferative DR was least common with a relative frequency of 2.25%. The calculated sensitivity was 80.3% and the specificity was 92.3%.

The performance reported in the included studies is summarized in **(S1 Table).**

**Fig 2:**
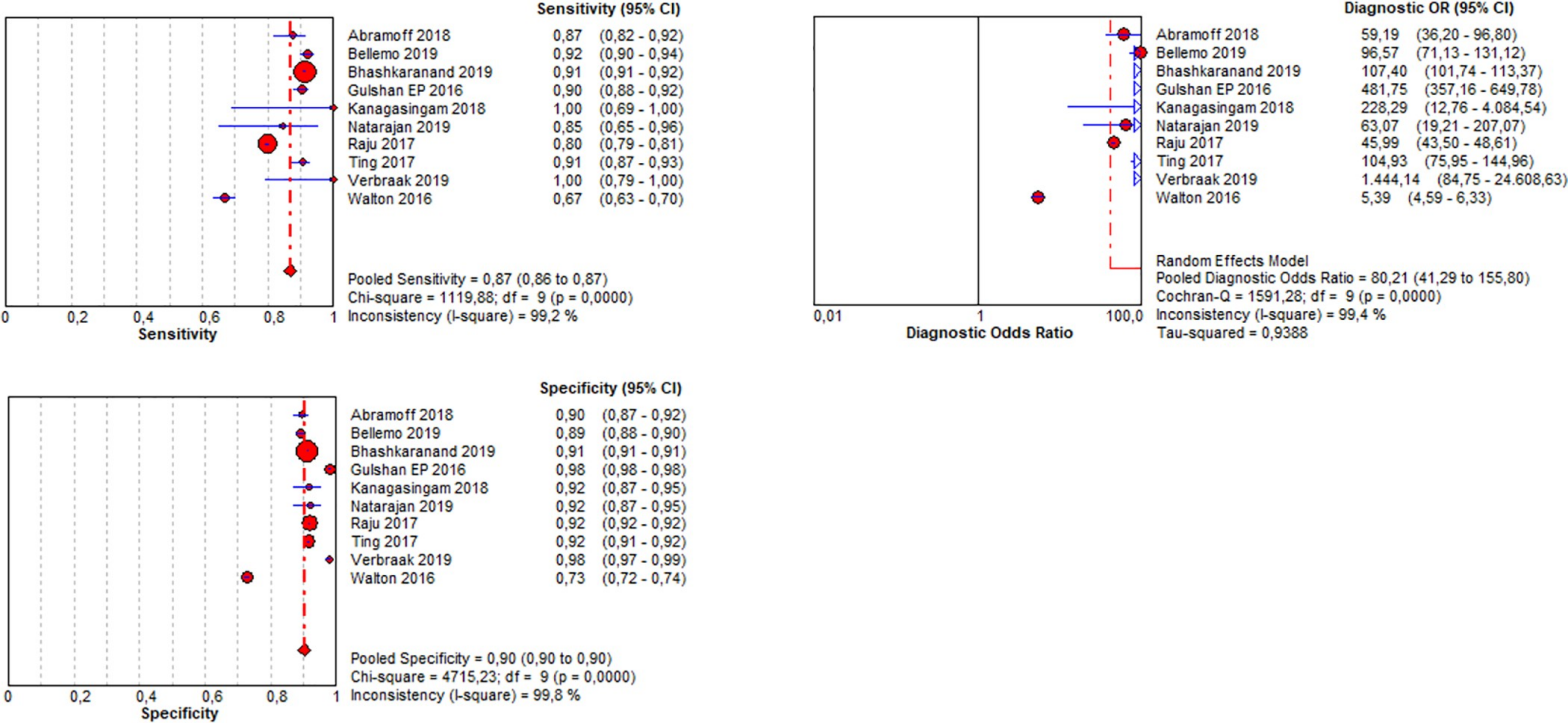
Pooled sensitivity, specificity and diagnostic odds ratio of any DR.

**Fig 3:**
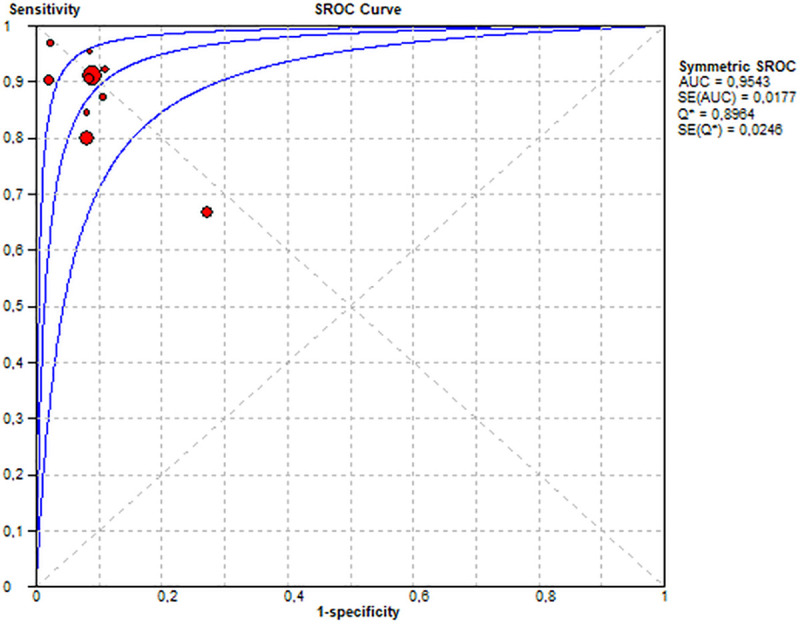
Summary receiver operating characteristic (SROC) curve.

In order to numerically compare the sensitivities and specificities calculated in the included studies, a meta-analysis was conducted, and forest plots were generated for those studies reporting the respective numbers (Figs 2 and 3).

**Fig 4:**
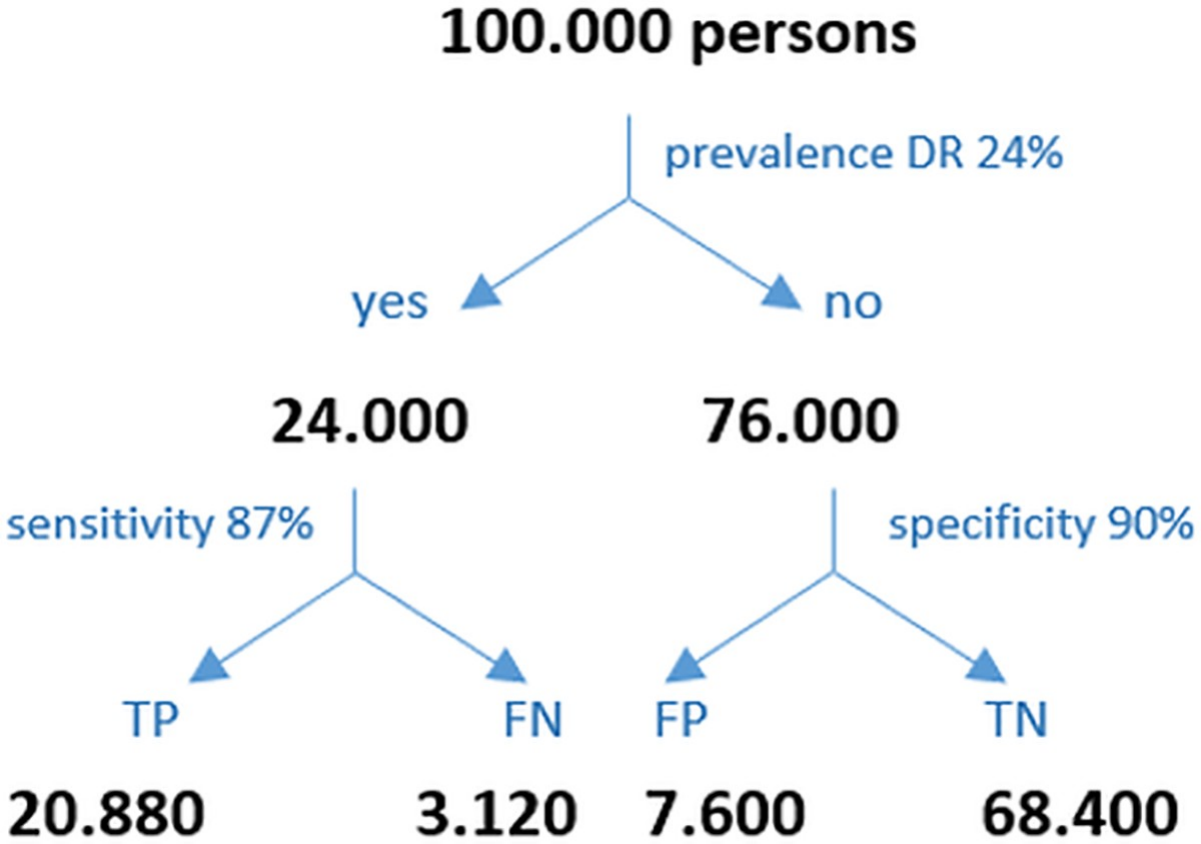
Positive and negative predictive values of DR for patients with diabetes type 1: (TP) true positive, (TN) true negative, (FP) false positive, (FN) false negative, (ppv) positive predictive value, (npv) negative predictive value. ppv = TP/(TP+FP) = 73% of all positively tested diabetes patients, 73% have a true DR and 27% do not. npv = TN/(TN+FN) = 95% of all negatively tested diabetes patients, 95% are truly negative and 5% are not.

The pooled sensitivity of all included studies was 87% and the pooled specificity was 90%. (Fig 2). The diagnostic performance may be interpreted for the PC setting as follows: In PC, the prevalence of patients with DM type 1 who have a DR amounts to 24% in Germany. Keeping this in mind, the PC provider must estimate the positive predicate value (ppv) in a given setting. The ppv and the negative predictive value (npv) may be calculated based on the prevalence, the sensitivity and the specificity (Fig 4). Accepted a prevalence of 24%, the calculated ppv is 73% and the calculated npv is 95%. Therefore 73% of the positively tested patients do in fact have a DR, and 95% of the negatively tested patients have truly no DR.

**Fig 5:**
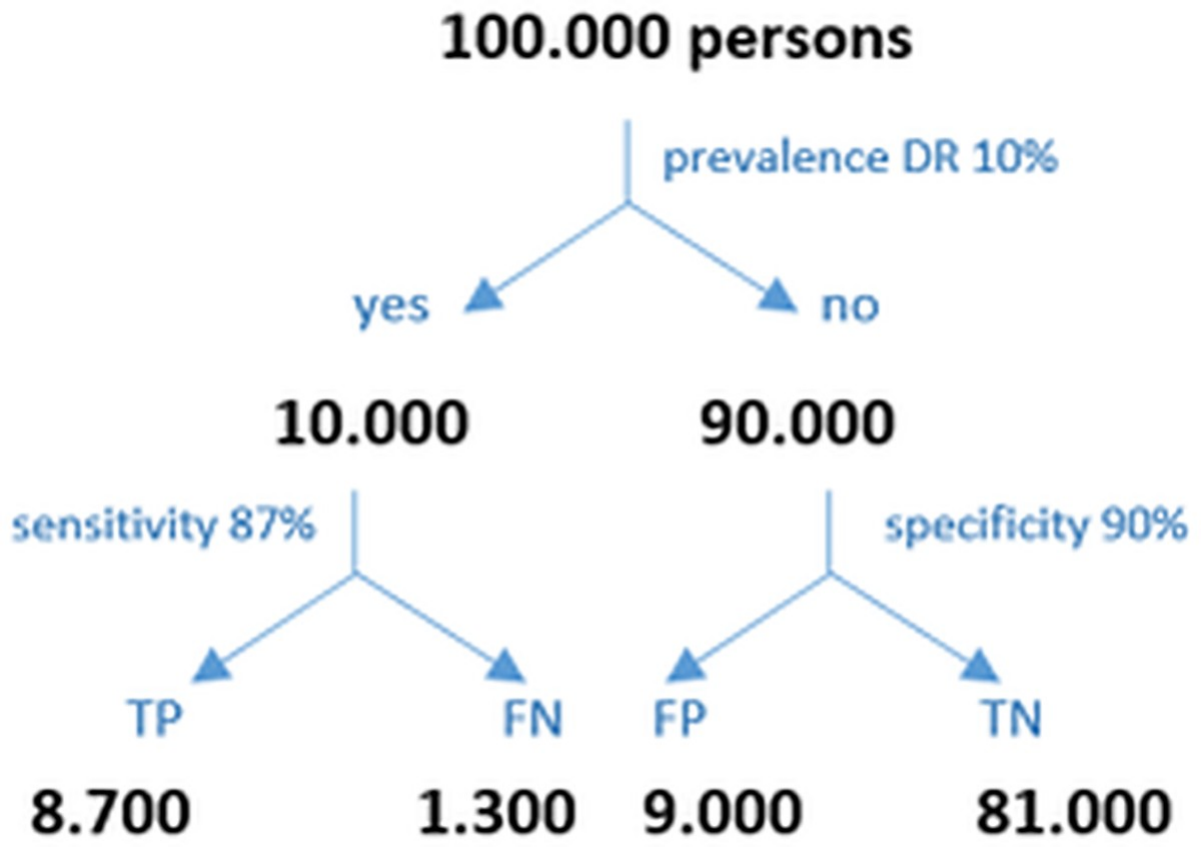
Positive and negative predictive values of DR for patients with diabetes type 2: (TP) true positive, (TN) true negative, (FP) false positive, (FN) false negative, (ppv) positive predictive value, (npv) negative predictive value. ppv = TP/(TP+FP) = 49% of all positively tested diabetes patients, 49% have a true DR and 51% do not. npv = TN/(TN+FN) = 98% of all negatively tested diabetes patients, 98% are truly negative for DR, while 2% are not.

Similarly, the prevalence of patients with type 2 DM who have a DR is 10% in the PC setting in Germany. The calculated ppv is 49% and the calculated npv is 98% (Fig 5). Accordingly, 49% of the positively tested patients do in fact have a DR, and 98% of the negatively tested patients have truly no DR.

### Quality of included studies

All studies applied an appropriate reference test to all patients, employed a reference standard that was independent of the index test, and described the protocol of the index test in detail. However, the physicians that acting as a reference standard are not validated and have an unknown relationship to clinical prognosis or outcome. Moreover, some studies insufficiently described inclusion and exclusion criteria, an appropriate time frame between reference test and index test to allow for an accurate evaluation, and detailed description of the reference standard. Furthermore, the study population was diverse in terms of ethnicity.

The detailed results of the QUADAS evaluation are listed in **(S2 Table).**

**Fig 6:**
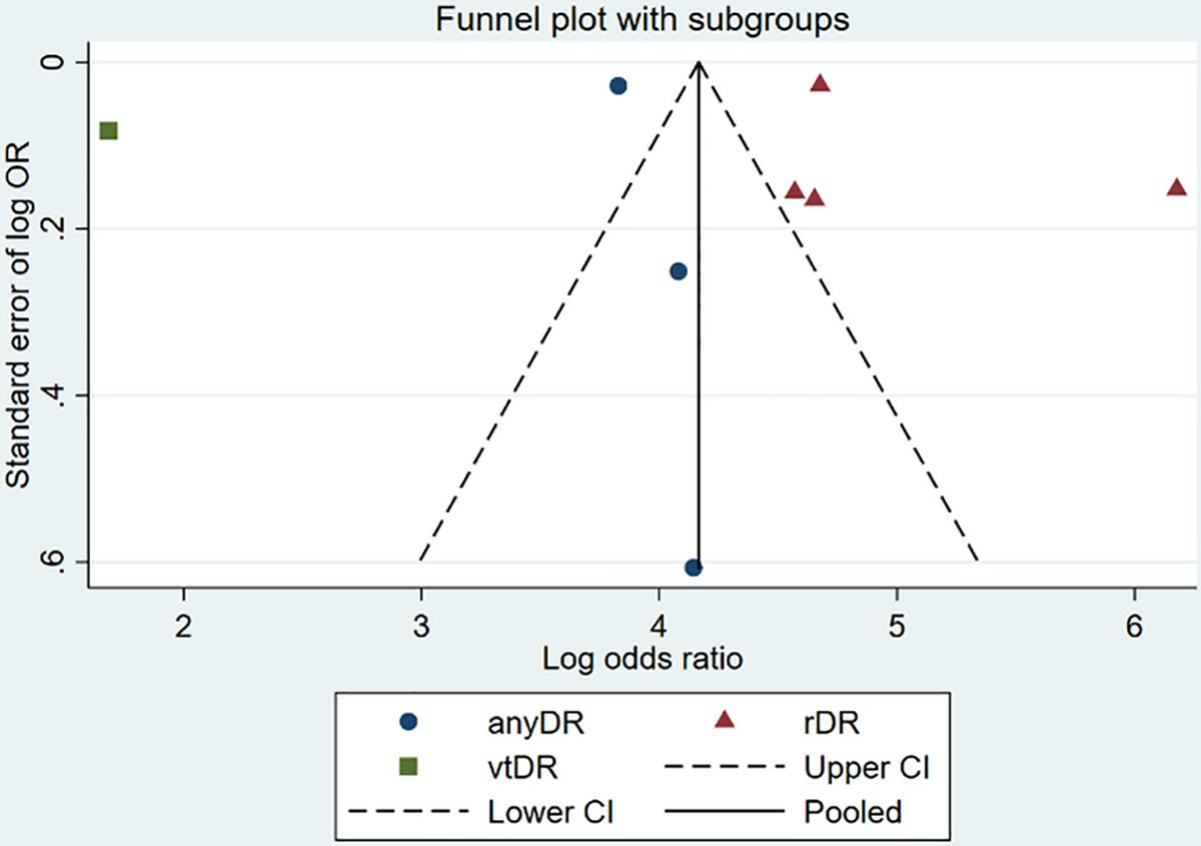
Funnel plot to assess publication bias.

Fig 6 shows the funnel plot to assess the bias in the individual studies, demonstrating an asymmetric distribution.

## Discussion

The aim of this meta-analysis was to determine the diagnostic performance of DL-based screening methods for DR in PC. One of the major findings of this study is that research on AI for the screening of diabetic retinopathy, especially in a primary care setting, does exist. However, the number of studies in this field is still scarce.

The sensitivity and specificity in the included studies were overall high, particularly for the detection of rDR. The FDA issued a cut-off value for the sensitivity and specificity of 85% and 82.5%, respectively [27]. Most of the studies assessed in this meta-analysis calculated a sensitivity and specificity above these reference values. Nevertheless, diagnostic performance has always to be evaluated with regard to the prevalence. With identical sensitivity and specificity values, the ppv is significantly lower in a low prevalence situation like in primary care than in a high prevalence situation like tertiary care.

Therefore, a positive test result may be of very different significance depending on the setting. In all studies included, the reference standard of the DL-performance were human specialists and solely the performance of the algorithms has been tested. However, the clinicians’, interpretation of fundus images has not been validated. Therefore, their sensitivity, specificity, presence or absence of racial, ethnic and other biases are unknown. The intergrader agreement between the grader’s specialists were mentioned only in two of ten studies, Natarajan et al. and Bhaskaranand et al. An excellent prognostic standard could be the usage of the ETDRS severity scale applied by a standardized reading center, such as the Wisconsin Reading Center, using 7-field or 4-widefield stereoscopic photographs. If not thoroughly validated, there is evidence that artificial intelligence has reached a level where it could even outperform doctors. A study, conducted in tertiary care setting, which compared the performance of DL-algorithm with that of human graders (international retinal specialists) attributed a higher sensitivity to the algorithms in comparison with human graders (97% vs. 74%) in detecting referable DR, while specificity was slightly below that of human graders (96% vs.98%) [28].

Another approach of reliably diagnosing diabetic retinopathy, especially diabetic macular edema, is by Optical Coherence Tomography (OCT). Even though the diagnostic performance for macular edema with OCT is higher than of fundus photographs [29, 30], it has not yet found its way into first line screening. However, in primary care, a simpler, less costly and time-consuming alternative seems more appropriate.

Applicability from the physician’s perspective: In principle, DL screening tools may offer novel diagnostic strategies for the detection of DR in PC. However, differences in the patient population, use of different thresholds to determine a positive test result, and the varying probability to accurately detect a DR do not allow for a general recommendation to use these tools in the clinical practice at this point.

However, the idea that eye screenings could be performed by general practitioners is intriguing and the fact that DL screening tools may be easily learned by non-ophthalmologist practitioners seems promising.

Applicability from the patient’s perspective: Appropriate eye care of patients with DM can be hindered e.g. by limited access to an eye care specialist due to remote living situations. In addition, patients’ adherence to the recommended screening schedule is low and results in late detection of a DR and limited therapeutic options. The relationship between the PC provider and the patient is also of importance in this context, as patients may feel more familiar with their family doctor than with a specialist who they have not been in contact with before. Of the studies shown here, several authors conclude that adherence to regular screenings may be enhanced if the PC physician is able to conduct them [18]. Patients with DR may be distinguished from those who need further regular screenings at the PC office [31]. If the access to regular screenings is facilitated with artificial intelligence technologies that allow for an initial diagnosis by the PC physician, screening rates may increase and referrals may be conducted more selectively [22].

Applicability from the society’s perspective: According to the current state of knowledge, it is not known whether the application of DL screening tools can in fact optimize the DR screening process and reduce the associated costs. It is, however, known that early detection of DR allows for an effective therapy and cost savings due to prevention of complications and blindness that occur in patients with an advanced disease [32]. If tedious manual screenings of fundus images could be replaced by DL technology screening methods, it is feasible to assume that human resources costs could be decreased and the efficiency of the screening process enhanced [33]. Further cost savings may be achieved by replacing conventional fundus cameras, which are expensive and require time for their correct use, by more efficient screening tools [34]. In addition, unnecessary referrals to ophthalmologists and the costs associated with specialist visits could be prevented by regular and early screenings in the PC office.

Tufail et al. (2017) described that ethnicity, gender or the type of camera did not affect the sensitivity or specificity of an automated eye screening with the EyeArt system [35]. The variability in the patient characteristics and selection criteria was however high in the studies assessed in our meta-analysis and included patients with type 1 and type 2 DM, hence it may not be concluded whether the DL tools are equally suitable for patients with both DM types. The intelligence of a DL system is only partial, as it diagnoses only what it was trained to detect, while a physician has a broader perspective integrating the individual patient characteristics. For an effective use of a DL algorithm, the sample must be representative for the patient population and should be based on an appropriate training set of high quality. This point must be stressed, since the selection of a representative patient sample for an accurate diagnosis must incorporate both legal aspects pertaining to patient data and patient criteria reflecting the target population.

Limitations: To the best of our knowledge, this is the first meta-analysis to evaluate the applicability of a DL based screening test for DR in PC. Currently available reviews [36, 37] do not consider PC nor the crucial aspect of prevalence.

To minimize bias, the whole screening and data extraction was conducted by two independent researchers. However, it must be acknowledged that the studies included in the present analysis were very distinct and the study quality was heterogenous.

One limitation of our analysis may be the presence of statistical heterogeneity. Meta-analysis was nonetheless deemed suitable as they reported the same DL interventions on patients with the same underlying disease. The aim of the meta-analysis was to identify all available studies in the field that met predefined inclusion and exclusion criteria and use this information to identify research gaps and provide suggestions for future improvement of studies in the field. Another limitation may be the lack of appropriate studies on AI in diabetic retinopathy screening in primary care. Therefore, the applicability of the results obtained in the meta-analysis is limited. In addition, the included studies lack a preregistration by which the hypothesis to be tested is made publicly available. Therefore, the safety and lack of bias may not be proven for these studies and evidence for their replicability is limited. The quality of the available studies is further diminished by lack of comparison of the clinician readings to prognostic standards, which is imperative for the evaluation of their sensitivity and specificity and potential observer variability and bias.

Furthermore, it must be acknowledged, that we also included retrospective studies assessing DL systems with available routine data. These studies might have confounding effects e.g. missing information on included type of patients and comorbidities of the patients. The included studies showed a wide range in the quality and quantity of the data used to train and validate the DL systems, which aggravates a direct comparison of the outcomes. Data obtained using publicly available datasets lack an evaluation of such systems in a real-life setting such as a retinopathy screening program. Moreover, the integrity of the AI algorithms employed in the studies and hence their external validity was limited by lack of a lock of the algorithm at the initiation of data interpretation. A direct comparison of the available studies is further hindered using multiple different camera systems with varying quality. The applicability to the general population is also difficult to determine, as the patient population included in many studies was heterogeneous and not representative of different patients participating in regular retinopathy screenings. The asymmetric distribution in the funnel plot may be due to a true publication bias resulting from preferred publication of larger studies showing significant positive outcomes over smaller studies with negative outcomes. It is feasible to assume that in the present study, such an asymmetric distribution stems from the observed large heterogeneity between the studies.

## Conclusion

Despite overall high values for the diagnostic performance of DL-algorithms, the different prevalence of patients with DR depending on e.g. the type of diabetes or the health care sector must be taken into consideration when interpreting a test result. With a pooled sensitivity of 87% and specificity of 90% screening for DR in patients with DM Type 2 in PC may be falsified with a probability of 98% whereas the positive predictive value is 49%.

However, especially in remote areas, this approach may still improve quality of care.

The currently available studies on this topic reveal multiple weaknesses and research gaps. More research is needed in form of prospective clinical studies demonstrating safety, efficacy, and equity, as well as presence or absence of racial, age, sex and ethnic bias. Suitable prognostic standards such as the ETDRS severity scale are available and should be applied by standardized reading centers to ensure a valid reference standard. A concise study design and study protocol including the best available prognostic and AI tools would enhance the applicability of the obtained data to clinical practice.

## Supporting information

S1 Checklist PRISMA 2009 checklist. (DOC)

S1 Table Data extraction table. (XLSX)

S2 Table QUADAS. (XLSX)

## Abbreviations

AUC: Area Under the Curve
DM: Diabetes Mellitus
DR: Diabetic Retinopathy
EDTRS: Early Treatment of Diabetic Retinopathy Study
IDF: International Diabetes Federation
npv: negative predictive value
ppv: positive predictive value
PC: Primary Care
rDR: referable diabetic retinopathy
vtDR: vision-threatening diabetic retinopathy
TP: true positive
TN: true negative
FP: false positive
FN: false negative

## References

[1] IDF. IDF Diabetes Atlas. 8th ed. 2017.

[2] BVA/DOG. Leitinie Nr.20 Diabetische Retinopathie. 2011.

[3] Voigt M, Schmidt S, Lehmann T, Köhler B, Kloos C, Voigt UA, et al. Prevalence and Progression Rate of Diabetic Retinopathy in Type 2 Diabetes Patients in Correlation with the Duration of Diabetes. Exp Clin Endocrinol Diabetes. 2018;126(9):570–6. 29183104 10.1055/s-0043-120570

[4] Simó-Servat O, Ciudin A, Ortiz-Zúñiga Á M, Hernández C, Simó R. Usefulness of Eye Fixation Assessment for Identifying Type 2 Diabetic Subjects at Risk of Dementia. J Clin Med. 2019;8(1):59. doi: 10.3390/jcm8010059 PMC6352169 30626106

[5] Ferris F L, Patz A, Macular edema. A complication of diabetic retinopathy. Survey of Ophthalmology, 28 452–461. 1984 6379946 10.1016/0039-6257(84)90227-3

[6] Garg S, Davis RM. Diabetic Retinopathy Screening Update. Clinical Diabetes. 2009;27(4):140–5. doi: 10.2337/diaclin.27.4.140

[7] Bundesärztekammer. Nationale Versorgungs Leitlinie (NVL) 2019 [Available from: https://www.leitlinien.de/nvl/html/netzhautkomplikationen/kapitel-1

[8] Hammes H-P, Lemmen KD, Bertram B. Diabetische Retinopathie und Makulopathie. Der Diabetologe. 2019;15(5):426–31.

[9] Bertram B, Gante C, Hilgers RD. Zunahme der Untersuchungen wegen Katarakt, Glaukom, diabetischer Retinopathie und Makuladegeneration. Der Ophthalmologe. 2014;111(8):757–64. 24343245 10.1007/s00347-013-2966-z

[10] Scherer M, Abholz H, Chenot J-F, Gerlach F, Kochen M. Versorgungsforschung in der Allgemeinmedizin und Familienmedizin. 2010.

[11] Poole D, Mackworth A, Goebel R. Computational Intelligence: A Logical Approach. 1998.

[12] Bini SA. Artificial Intelligence, Machine Learning, Deep Learning, and Cognitive Computing: What Do These Terms Mean and How Will They Impact Health Care? J Arthroplasty. 2018;33(8):2358–61. 29656964 10.1016/j.arth.2018.02.067

[13] Kurc T, Bakas S, Ren X, Bagari A, Momeni A, Huang Y, et al. Segmentation and Classification in Digital Pathology for Glioma Research: Challenges and Deep Learning Approaches. Front Neurosci. 2020;14:27. 32153349 10.3389/fnins.2020.00027 PMC7046596

[14] Geras KJ, Mann RM, Moy L. Artificial Intelligence for Mammography and Digital Breast Tomosynthesis: Current Concepts and Future Perspectives. Radiology. 2019;293(2):246–59. 31549948 10.1148/radiol.2019182627 PMC6822772

[15] McInnes MDF, Moher D, Thombs BD, McGrath TA, Bossuyt PM, Clifford T, et al. Preferred Reporting Items for a Systematic Review and Meta-analysis of Diagnostic Test Accuracy Studies: The PRISMA-DTA Statement. Jama. 2018;319(4):388–96. 29362800 10.1001/jama.2017.19163

[16] Richardson WS, Wilson MC, Nishikawa J, Hayward RS. The well-built clinical question: a key to evidence-based decisions. ACP J Club. 1995;123(3):A12–3. 10.7326/ACPJC-1995-123-3-A12 7582737

[17] Whiting P, Rutjes AW, Reitsma JB, Bossuyt PM, Kleijnen J. The development of QUADAS: a tool for the quality assessment of studies of diagnostic accuracy included in systematic reviews. BMC Med Res Methodol. 2003;3:25. 14606960 10.1186/1471-2288-3-25 PMC305345

[18] Abràmoff MD, Lavin PT, Birch M, Shah N, Folk JC. Pivotal trial of an autonomous AI-based diagnostic system for detection of diabetic retinopathy in primary care offices. NPJ Digit Med. 2018;1:39. 31304320 10.1038/s41746-018-0040-6 PMC6550188

[19] Natarajan S, Jain A, Krishnan R, Rogye A, Sivaprasad S. Diagnostic Accuracy of Community-Based Diabetic Retinopathy Screening With an Offline Artificial Intelligence System on a Smartphone. JAMA Ophthalmol. 2019;137(10):1182–8. 31393538 10.1001/jamaophthalmol.2019.2923 PMC6692680

[20] Verbraak FD, Abramoff MD, Bausch GCF, Klaver C, Nijpels G, Schlingemann RO, et al. Diagnostic Accuracy of a Device for the Automated Detection of Diabetic Retinopathy in a Primary Care Setting. Diabetes Care. 2019;42(4):651–6. 30765436 10.2337/dc18-0148

[21] Walton OBt, Garoon RB, Weng CY, Gross J, Young AK, Camero KA, et al. Evaluation of Automated Teleretinal Screening Program for Diabetic Retinopathy. JAMA Ophthalmol. 2016;134(2):204–9. 26720694 10.1001/jamaophthalmol.2015.5083

[22] Bhaskaranand M, Ramachandra C, Bhat S, Cuadros J, Nittala MG, Sadda SR, et al. The Value of Automated Diabetic Retinopathy Screening with the EyeArt System: A Study of More Than 100,000 Consecutive Encounters from People with Diabetes. Diabetes Technol Ther. 2019;21(11):635–43. 31335200 10.1089/dia.2019.0164 PMC6812728

[23] Kanagasingam Y, Xiao D, Vignarajan J, Preetham A, Tay-Kearney ML, Mehrotra A. Evaluation of Artificial Intelligence-Based Grading of Diabetic Retinopathy in Primary Care. JAMA Netw Open. 2018;1(5):e182665. 30646178 10.1001/jamanetworkopen.2018.2665 PMC6324474

[24] Ting DSW, Cheung CY, Lim G, Tan GSW, Quang ND, Gan A, et al. Development and Validation of a Deep Learning System for Diabetic Retinopathy and Related Eye Diseases Using Retinal Images From Multiethnic Populations With Diabetes. Jama. 2017;318(22):2211–23. 29234807 10.1001/jama.2017.18152 PMC5820739

[25] Gulshan V, Peng L, Coram M, Stumpe MC, Wu D, Narayanaswamy A, et al. Development and Validation of a Deep Learning Algorithm for Detection of Diabetic Retinopathy in Retinal Fundus Photographs. Jama. 2016;316(22):2402–10. 27898976 10.1001/jama.2016.17216

[26] Raju M, Pagidimarri V, Barreto R, Kadam A, Kasivajjala V, Aswath A. Development of a Deep Learning Algorithm for Automatic Diagnosis of Diabetic Retinopathy. Stud Health Technol Inform. 2017;245:559–63. 29295157

[27] Schmidt-Erfurth U, Sadeghipour A, Gerendas BS, Waldstein SM, Bogunović H. Artificial intelligence in retina. Prog Retin Eye Res. 2018;67:1–29. doi: 10.1016/j.preteyeres.2018.07.004 30076935

[28] Ruamviboonsuk P, Krause J, Chotcomwongse P, Sayres R, Raman R, Widner K, et al. Deep learning versus human graders for classifying diabetic retinopathy severity in a nationwide screening program. npj Digital Medicine. 2019;2(1):25. 31304372 10.1038/s41746-019-0099-8 PMC6550283

[29] Wang YT, Tadarati M, Wolfson Y, Bressler SB, Bressler NM. Comparison of Prevalence of Diabetic Macular Edema Based on Monocular Fundus Photography vs Optical Coherence Tomography. JAMA Ophthalmol 2016; 134:222–228. 2016 doi: 10.1001/jamaophthalmol.2015.5332 26719967

[30] Virgili G, Menchini F., Casazza G., Hogg R. Radha R D, Wang X, et al. Optical coherence tomography (OCT) for detection of macular oedema in patients with diabetic retinopathy. Cochrane Database Syst Rev. 2015 10.1002/14651858.CD008081.pub3 PMC4438571 25564068

[31] Abràmoff MD, Lou Y, Erginay A, Clarida W, Amelon R, Folk JC, et al. Improved Automated Detection of Diabetic Retinopathy on a Publicly Available Dataset Through Integration of Deep Learning. Invest Ophthalmol Vis Sci. 2016;57(13):5200–6. 27701631 10.1167/iovs.16-19964

[32] Klonoff DC, Schwartz DM. An economic analysis of interventions for diabetes. Diabetes Care. 2000;23(3):390–404. 10868871 10.2337/diacare.23.3.390

[33] Nørgaard MF, Grauslund J. Automated Screening for Diabetic Retinopathy—A Systematic Review. Ophthalmic Res. 2018;60(1):9–17. 29339646 10.1159/000486284

[34] Bawankar P, Shanbhag N, K SS, Dhawan B, Palsule A, Kumar D, et al. Sensitivity and specificity of automated analysis of single-field non-mydriatic fundus photographs by Bosch DR Algorithm-Comparison with mydriatic fundus photography (ETDRS) for screening in undiagnosed diabetic retinopathy. PLoS One. 2017;12(12):e0189854. 29281690 10.1371/journal.pone.0189854 PMC5744962

[35] Tufail A, Rudisill C, Egan C, Kapetanakis VV, Salas-Vega S, Owen CG, et al. Automated Diabetic Retinopathy Image Assessment Software: Diagnostic Accuracy and Cost-Effectiveness Compared with Human Graders. Ophthalmology. 2017;124(3):343–51. 28024825 10.1016/j.ophtha.2016.11.014

[36] Islam Md M, Yang H Ch, Poly T N, Jian W-S, Li Yu-C, et al. Deep learning algorithms for detection of diabetic retinopathy in retinal fundus photographs: A systematic review and meta-analysis. Elsevier. 2020 0169–2607 10.1016/j.cmpb.2020.105320 32088490

[37] Nielsen K B, Lautrup M L, Andersen J K.H, Savarimuthu T.R, Graslund J, et al. Deep Learning-based Algorithms in Screening of Diabetic Retinopathy: A Systematic Review of Diagnostic Performance. Ophthalmology Retina. 2018 10.1016/j.oret.2018.10.014 31014679

